# Evaluation of the combined PBL-CBL teaching method based on Thorndike’s Learning Theory in the education of bacterial resistance detection

**DOI:** 10.3389/fmed.2026.1897289

**Published:** 2026-08-25

**Authors:** Yifeng Liu, Cuiju Mo, Meng Li

**Affiliations:** 1 Department of Clinical Laboratory, The First Affiliated Hospital of Guangxi Medical University, Nanning, China; 2 Teaching and Research Section of Clinical Laboratory Science, The First School of Clinical Medicine, Guangxi Medical University, Nanning, China

**Keywords:** bacterial resistance detection, case-based learning, problem-based learning, teaching effect, Thorndike’s Learning Theory

## Abstract

**Background:**

Bacterial resistance detection is a core skill for microbiology technicians. Given the limitations of traditional teaching, exploring an efficient novel method is crucial for improving medical interns’ training quality in this field. This study investigates the effects of the combined PBL-CBL teaching method guided by Thorndike’s Learning Theory on bacterial resistance detection education.

**Methods:**

Medical interns from the Microbiology Section, Department of Laboratory Medicine, The First Affiliated Hospital of Guangxi Medical University, from August 2024 to August 2025, were enrolled and then divided into a control group (receiving the traditional teaching method) and an experimental group (receiving the combined teaching method). Pre- and post-intervention comparisons included professional knowledge scores, critical thinking (measured by the CTDI-CV scale), learning fatigue (measured by the MBI scale), and teaching effect evaluations.

**Results:**

The experimental group outperformed the control group in theoretical knowledge, practical skills, and antimicrobial susceptibility report analysis. They also showed stronger cognitive levels (curiosity, open-mindedness, systematic and analytical thinking) with higher CTDI-CV scores, lower emotional exhaustion but higher personal accomplishment on the MBI scale, and better teaching effect evaluations, particularly in the aspects of highlighting key and difficult points, promoting active learning, and enhancing clinical practice skills. All differences were statistically significant.

**Conclusion:**

For education of bacterial resistance detection, the combined PBL-CBL teaching method guided by Thorndike’s Learning Theory can improve medical interns’ professional knowledge, cultivate critical thinking skills, reduce learning burnout, and eventually achieve favorable teaching outcomes.

## 1 Introduction

With the growing prominence of global public health concerns, bacterial resistance has become a critical challenge threatening human health. To effectively curb the spread of multidrug-resistant bacteria, building a talent team of medical laboratory professionals, equipped with solid microbiological knowledge, proficient experimental skills, and robust clinical thinking abilities, has become an urgent priority for safeguarding public health (1). As a core competency in Medical Laboratory Technology, the quality of education in bacterial resistance testing directly impacts whether future laboratory physicians can deliver accurate, timely antimicrobial susceptibility results and medication recommendations to support clinical anti-infective treatment (2, 3). Therefore, reforming and innovating the teaching methodologies in this area to enhance educational outcomes carries profound practical significance and considerable urgency.

At present, the traditional teaching of bacterial resistance detection mostly adopts a linear model, such as “theoretical teaching + experimental operation.” Although this model can deliver knowledge systematically, its spoon-feeding nature fails to foster students’ independent learning, clinical thinking, and abilities to learn by analogy. As a result, they still struggle to be competent in practical clinical work after training (4, 5). To address these limitations, modern pedagogical methods such as problem-based learning (PBL) and case-based learning (CBL) have been gradually introduced into medical education in recent years. PBL employs open-ended questions to stimulate students’ self-directed exploration and cultivate their ability to integrate knowledge and solve problems (6). CBL, on the other hand, uses real clinical cases to create immersive learning scenarios that enhance clinical thinking and decision-making skills (7). Numerous studies worldwide have demonstrated that a hybrid approach integrating both methods holds promise for achieving holistic development in “knowledge-skills-competency,” representing an ideal pathway to address challenges in clinical training (8–10). However, current reports about the effects of this method on the education of bacterial resistance detection remain limited in China.

According to Thorndike’s Learning Theory, learning is a process of continuous self-optimization through repeated trial and error and reinforcement. This theory provides a classical framework for constructing scientific instructional design (11). Here, we aim to use this theory as a core to systematically design and develop a combined PBL-CBL teaching model for the education of bacterial resistance detection. We also scientifically evaluate the effects of this model on improving intern medical students’ theoretical knowledge, experimental operation skills, clinical thinking abilities, and motivation for self-directed learning by comparative teaching practices. This research not only provides an effective and theoretically grounded teaching method for the education of bacterial resistance detection but also offers significant references and practical examples for deepening the reform in the education of Medical Laboratory Technology and cultivating high-quality application-oriented talents.

## 2 Materials and methods

### 2.1 Participants

**Figure 1:**
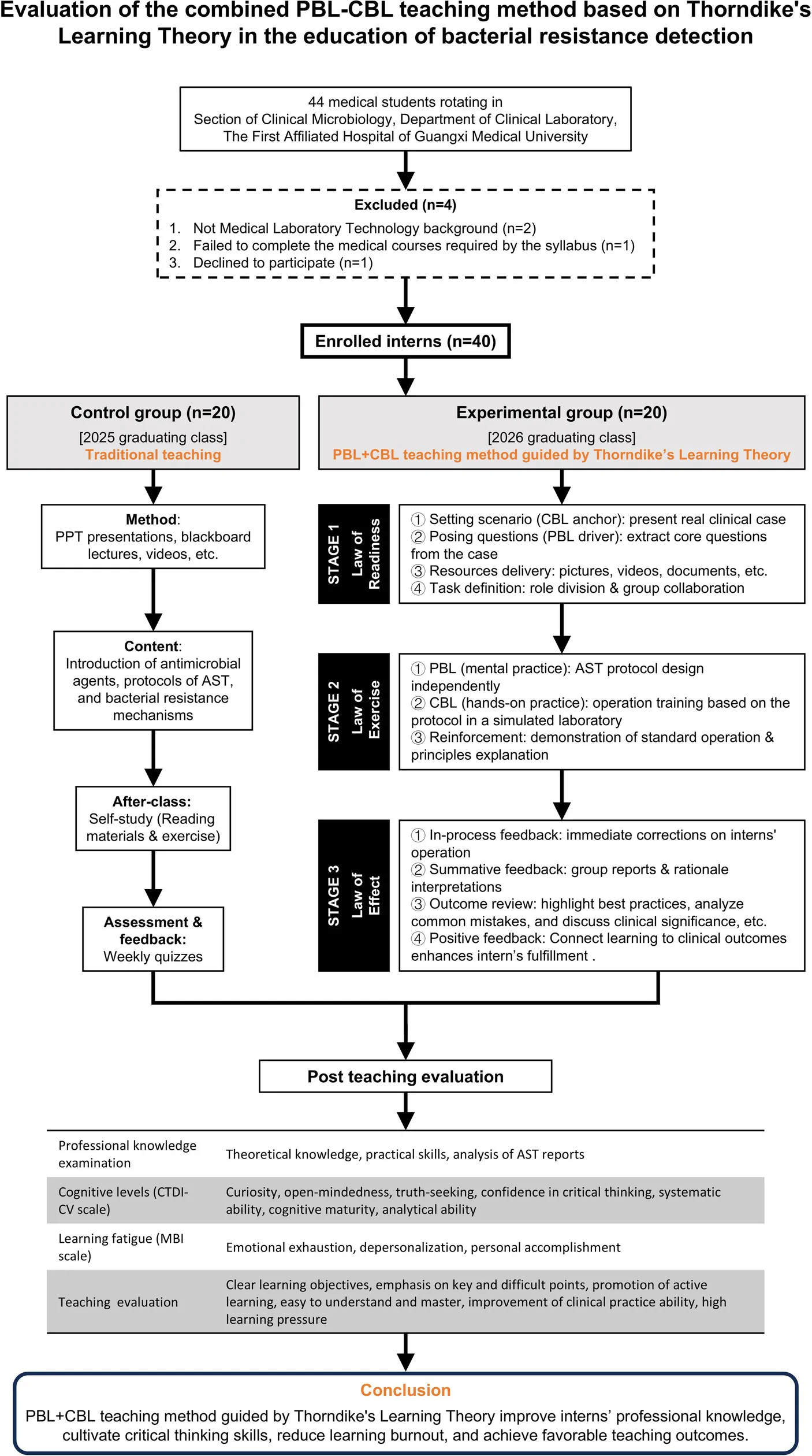
Flowchart illustrates the overall study design and detailed teaching procedures for both groups.

As shown in the flowchart (Figure 1), medical students who interned in the Section of Clinical Microbiology, Department of Clinical Laboratory, The First Affiliated Hospital of Guangxi Medical University, from August 2024 to August 2025 were selected as research subjects. Screening was conducted according to the following criteria:

Inclusion criteria: (1) Full-time undergraduate student with a background in Medical Laboratory Technology. (2) Having completed the medical courses required by the major’s syllabus and having basic theoretical knowledge of clinical microbiology. (3) In good physical and mental health, with no history of major diseases. (4) Voluntarily participating in this study, having good compliance, and having signed the informed consent form. Exclusion criteria: (1) Students who failed to complete the internship program in full due to personal reasons (e.g., taking leave for postgraduate entrance exams, transferring to other internship sites, etc.); (2) Students participating in multiple research projects simultaneously.

A total of 40 medical interns were ultimately enrolled and divided into two groups of 20 each according to the teaching method employed. Among them, 20 interns from the 2025 graduating class were assigned to the control group, which received the traditional teaching method; 20 interns from the 2026 graduating class were assigned to the experimental group, which received the combined PBL-CBL teaching method guided by Thorndike’s Learning Theory. The control group consisted of 7 males and 13 females, aged between 21 and 25 years, with a mean age of (23.05 ± 0.94) years. The experimental group consisted of 9 males and 11 females, aged between 21 and 25 years, with a mean age of (22.75 ± 1.02) years. No statistically significant differences were found in gender (*χ*^2^ = 0.417, *p* = 0.519) or age (*t* = 0.965, *p* = 0.340) between the two groups, indicating comparability. A 3-week rotation was implemented for the training, with two 2-h teaching sessions per week, providing each group with a total of 12 contact hours.

### 2.2 Study design

Medical interns from both groups were taught by the same three laboratory technicians, who had at least 10 years of work experience in clinical microbiology, demonstrated sound professional ethics and competence, and held an associate senior title or above. This was intentionally designed to eliminate the potential confounding effect of differences in teaching style, experience, or enthusiasm among different teachers.

To avoid cross-contamination of teaching methods that may occur if the same instructors deliver teaching for both groups, the following measures were implemented in this study: (1) The teaching protocols for the two groups were documented separately prior to the rotation, and instructors prepared lessons strictly in accordance with the established protocols; (2) Instructors were required to refrain from mentioning or demonstrating any case materials or teaching approaches adopted in the experimental group during lectures for the control group; (3) A researcher not involved in direct teaching was assigned to conduct unscheduled classroom inspections to monitor consistent implementation of teaching arrangements; (4) Teaching sessions for the two groups were scheduled at different time slots (control group first, followed by the experimental group) to minimize unintentional interference caused by instructors recalling content from both groups simultaneously.

Meanwhile, all enrolled interns needed to complete a baseline assessment within 1 week prior to the start of the clinical rotation. This baseline test covered professional knowledge, cognitive levels, and learning fatigue, with scoring rubrics identical to those described in Section 2.3 of this paper. This test and the following test used for outcome evaluation were designed to be equivalent in content coverage and difficulty, but the specific test items were completely different to avoid recall bias. Both tests were scored by the same three instructors using standardized scoring rubrics. Neither the instructors nor the interns were aware of the subsequent grouping at the time of the baseline assessment.

#### 2.2.1 Control group

In accordance with the syllabus for Clinical Microbiology Laboratory Technology, medical interns from this group received theoretical instruction, including the categories of antimicrobial agents, protocols of antimicrobial susceptibility testing (AST), and common bacterial resistance mechanisms. This instruction was delivered through various methods, such as PowerPoint presentations, blackboard lectures, and video supplements. Relevant reading materials and exercises were assigned after class, requiring students to review independently and complete them in time. Weekly quizzes regularly assessed learning effects, enabling timely identification of gaps and targeted guidance. Students were encouraged to finish the ASTs and interpret the susceptibility reports under the supervision of teachers.

#### 2.2.2 Experimental group

Medical interns from this group received the combined PBL-CBL teaching method guided by Thorndike’s Learning Theory.

(1) Thorndike’s Law of Readiness: (a) Creating a clinical scenario (CBL Anchor): Prior to the start of the session, a representative real-world clinical case was presented. For example, “Patient Zhang, a 68-year-old male, was admitted to the intensive care unit with severe pneumonia. Empirical treatment with ceftazidime initially led to clinical improvement. However, 3 days later, he developed recurrent high fever with a peak of 39 °C. Serum procalcitonin and C-reactive protein levels were elevated. Chest computed tomography revealed bilateral bronchial wall thickening, scattered patchy and nodular high-density opacities surrounding the bronchi, presenting as a “tree-in-bud” sign, along with a small amount of pleural effusion. Culture of bronchoalveolar lavage fluid yielded *Klebsiella pneumoniae* (*K. pneumoniae*), with a colony count exceeding 10^5^ CFU/mL. The antimicrobial susceptibility testing demonstrated that the isolate was resistant to amoxicillin-clavulanate, piperacillin-tazobactam, ceftriaxone, ceftazidime, ceftazidime-avibactam, cefepime, cefoxitin, aztreonam, imipenem, meropenem, and ertapenem. The patient remained on mechanical ventilation, with a declining trend in oxygenation index. How do you interpret these results? What should we do next to assist the clinicians?” (b) Posing core questions (PBL Driver): Teachers guided students to extract core questions from the clinical case. For example, “How can we confirm the resistance phenotype of this *K. pneumoniae* strain? Does it produce ESBLs or carbapenemases? What are the implications of enzyme detection results for guiding clinical antimicrobial therapy?” (c) Providing a learning package: Teachers provided pre-prepared learning resources, including *Clinical and Laboratory Standards Institute (CLSI) M100 Performance Standards for Antimicrobial Susceptibility Testing*, links to operation videos, reviews about drug resistance mechanisms, and expert consensus documents in the relevant field. (d) Defining the task: Students were informed of the final task for this unit. They were assigned to small groups with encouragement for internal role division. Then, they were required to execute a confirmatory AST for the strain isolated from the clinical case, submit the report, and try to draft medication recommendations for clinicians based on the AST results.

(2) Thorndike’s Law of Exercise: (a) Independent exploration and protocol design (PBL mental practice): Students were required to establish a protocol about AST of *K. pneumoniae*, which included method selection for ASTs [Kirby-Bauer (K-B) method, E-test, or broth microdilution (BMD) method?] and enzyme types detection (double-disk synergy test or enzyme inhibitor enhancement test?), reagents and equipment, specific operational procedures, result interpretation criteria, and key points of quality control. During this phase, teachers deepened students’ thinking by asking questions, such as “Why do you choose the double-disk synergy method for enzyme types detection?” and “What kind of strain will you choose for quality control?,” preventing them from falling into misunderstandings. (b) Practical operation training (CBL hands-on practice): Students proceeded to a simulated laboratory or actual clinical workstation to execute the protocol designed by their group, including preparing bacterial suspensions, placing the antibiotic disks, drug dilutions, and quality control. (c) Reinforcement: The key to this stage is repeated and standardized operations. Teachers required students not only to perform operations correctly but also to understand the principle behind each step (e.g., “Why must the bacterial suspension concentration be accurate?” “Why is sufficient incubation time required?”) to prevent the formation of incorrect operational habits.

(3) Thorndike’s Law of Effect: (a) In-process feedback: Teachers circulated throughout the laboratory, providing immediate comment on students’ techniques. For example, “Your preparation of the bacterial suspension meets the standard.” and “The flame sterilization step for the forceps was missed during disk placement. Please repeat this step.” This immediate feedback most effectively establishes the “Law of Effect.” (b) Summative feedback: Each group presented their reports and explained the rationale behind their interpretations. Teachers subsequently announced the AST result and patient’s outcome in the real clinical setting. For example, “The isolate was confirmed to produce ESBLs and metallo-beta-lactamase. The patient’s temperature decreased after the therapy was switched to ceftazidime/avibactam combined with aztreonam, based on the result of the final AST report.” (c) Outcome review: Teachers conducted a comparative analysis of the operational processes, result interpretations, and report standardization between the two groups. They not only focused on praising best practices and analyzing common mistakes but also systematically reviewed the operational flow and clinical significance of AST and enzyme type detection. (d) Professional fulfillment (positive feedback): Allowing students to witness the direct connection between their own learning gains (an accurate test report) and a good clinical prognosis (patient improvement) provided a strong sense of accomplishment and satisfaction. It greatly enhanced the effectiveness of the learning experience and helped students deeply understand the value of this skill.

### 2.3 Observation targets and evaluation methods

(1) Professional knowledge scores: The professional knowledge scores of medical interns from the two groups were compared before and after the teaching intervention. The total score comprises three components: theoretical knowledge, practical skills, and analysis of antimicrobial susceptibility reports. The assessment of theoretical knowledge was conducted via a closed-book examination consisting of single-choice questions, term explanations, fill-in-the-blank questions, and short-answer questions, with a maximum score of 100 points. The practical skills covered techniques such as Gram staining, isolation culture, inoculation, AST (K-B and BMD methods), and enzyme inhibitor enhancement test. The assessment was scored by teachers based on standardized scoring rubrics, with a maximum score of 100 points. In the section on analysis of antimicrobial susceptibility reports, students were required to start from the AST report, briefly describe the key points in the positive AST report review, analyze the underlying mechanisms of drug resistance in the detected pathogen, and provide appropriate medication recommendations to clinicians according to the case characteristics. Students randomly selected a report from a test bank and answered orally. The assessment was scored by teachers based on standardized scoring rubrics, with a maximum score of 100 points.

(2) Cognitive levels: The Critical Thinking Disposition Inventory-Chinese Version (CTDI-CV) was completed anonymously to compare the cognitive levels of medical interns from the two groups before and after the teaching intervention. The total score consists of seven dimensions: curiosity, open-mindedness, truth-seeking, confidence in critical thinking, systematic ability, cognitive maturity, and analytical ability. Each dimension contains 10 items scored on a scale of 1–6 points. The maximum score for a single dimension is 60 points, and the total maximum score of the CTDI-CV scale is 420 points. A score of ≥40 points for an individual dimension indicates a positive disposition in that trait. A total score of ≥280 points suggests the presence of overall critical thinking ability. A score of ≥40 in a single dimension indicates a positive trait in that dimension. A total score of ≥280 suggests the presence of overall critical thinking ability (12).

(3) Learning fatigue: Learning fatigue among the medical interns in both groups was assessed and compared after the teaching intervention using the anonymous Maslach Burnout Inventory (MBI) scale. This scale comprises three dimensions: emotional exhaustion, depersonalization, and personal accomplishment, containing 9, 5, and 8 items, respectively. The first two dimensions adopt forward scoring, with higher scores indicating greater burnout, and each item is scored from 0 to 6 points. The last dimension employs reverse scoring, where lower scores correspond to more severe job burnout (13).

(4) Evaluation of teaching effects: A self-developed questionnaire of teaching effects evaluation was administered anonymously to compare the recognition of the respective teaching methods between the two groups of medical interns after the teaching intervention (Supplementary Table 1). The questionnaire was developed by the research team on the basis of the intended learning outcomes of the bacterial antimicrobial resistance testing practicum and the educational objectives of the PBL + CBL teaching intervention. The wording and relevance of the items were reviewed by three faculty members with expertise in clinical microbiology, medical laboratory education, and clinical teaching before use. The questionnaire assessed the following items: clear learning objectives, emphasis on key and difficult points, promotion of active learning, easy to understand and master, enhancement of clinical practice ability, and high learning pressure. Each item was rated on a 5-point scale, ranging from 1 (“strongly disagree”) to 5 (“strongly agree”). Items 1–5 were positively worded, with higher scores indicating more favorable perceptions of the teaching approach. Item 6 assessed perceived learning pressure and was negatively oriented; thus, a higher score indicated greater perceived learning pressure. The composite score was calculated as the sum of items 1–5 minus the score for item 6, yielding a possible score range of 0–24. Higher composite scores indicated more favorable overall acceptance of the teaching method, taking learning pressure into account.

To validate the instrument, we assessed its content validity by inviting three senior clinical educators to review the item relevance; the item-level content validity index (I-CVI) ranged from 0.83 to 1.00, and the average scale-level CVI (S-CVI/Ave) was 0.92. Construct validity was examined via confirmatory factor analysis on the total sample, which yielded a single-factor structure with factor loadings from 0.55 to 0.82 and adequate model fit (CFI = 0.95, RMSEA = 0.07). Internal consistency was satisfactory, with a Cronbach’s α coefficient of 0.79 for the six items. These results support the questionnaire’s reliability and validity for this study.

### 2.4 Statistical analysis

The Shapiro–Wilk test and Levene’s test were employed to assess the normality of distribution and homogeneity of variances, respectively (Supplementary Table 2). For continuous data, if the data followed a normal distribution, they were expressed as mean ± standard deviation, and the independent samples *t*-test was used for inter-group comparisons. If the data did not conform to a normal distribution, they were presented as median (lower quartile, upper quartile), and the Mann–Whitney U test was applied for inter-group comparisons. For categorical data, the *χ*^2^ test was used for inter-group comparisons. All statistical analyzes were performed using SPSS 25.0 (IBM Corp., Armonk, NY, USA). For all tests, a *p* value of <0.05 was considered statistically significant.

## 3 Results

### 3.1 The combined PBL-CBL teaching method guided by Thorndike’s Learning Theory improves the professional knowledge of medical interns

**Figure 2:**
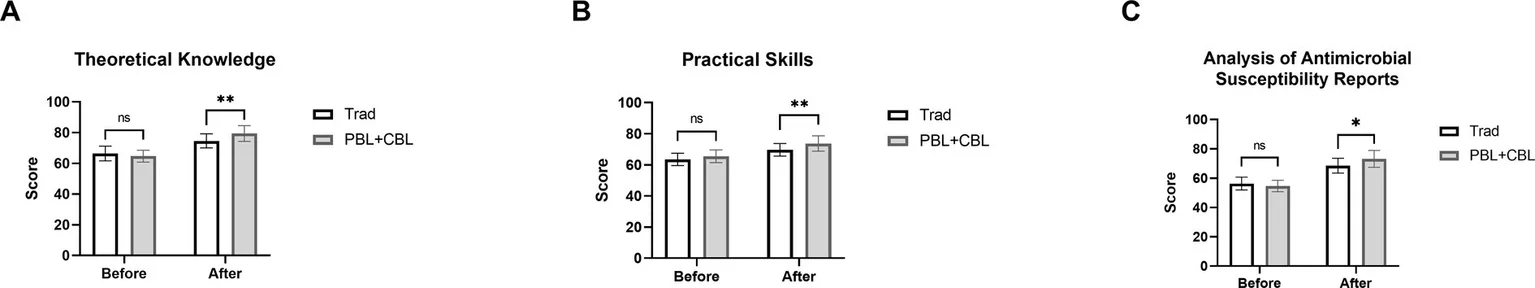
Comparison of professional knowledge scores between two groups of medical interns before and after teaching intervention. Observation indicators included scores in **(A)** theoretical knowledge, **(B)** practical skills, and **(C)** analysis of antimicrobial susceptibility reports. Statistical analysis was performed using Student’s *t*-test. All data are presented as mean ± standard deviation. *, *p* < 0.05; **, *p* < 0.01; ns, not significant; Trad, traditional teaching method in control group; PBL + CBL, combined PBL-CBL teaching method guided by Thorndike’s Learning Theory in experimental group.

As shown in Figure 2, prior to the teaching intervention, no statistically significant differences were observed between the two groups of students in the assessments of theoretical knowledge (*t* = 1.232, *p* = 0.225), practical skills (*t* = −1.613, *p* = 0.115), and analysis of antimicrobial susceptibility reports (*t* = 1.301, *p* = 0.201). However, after the teaching intervention, students in the experimental group got higher scores of theoretical knowledge (*t* = −3.134, *p* = 0.003), practical skills (*t* = −2.806, *p* = 0.008), and analysis of antimicrobial susceptibility reports (*t* = −2.644, *p* = 0.012) compared to those in the control group. All these differences were statistically significant.

### 3.2 The combined PBL-CBL teaching method guided by Thorndike’s Learning Theory broadens the perspectives, enhances the curiosity, systematic, and analytical abilities of medical interns

**Figure 3:**
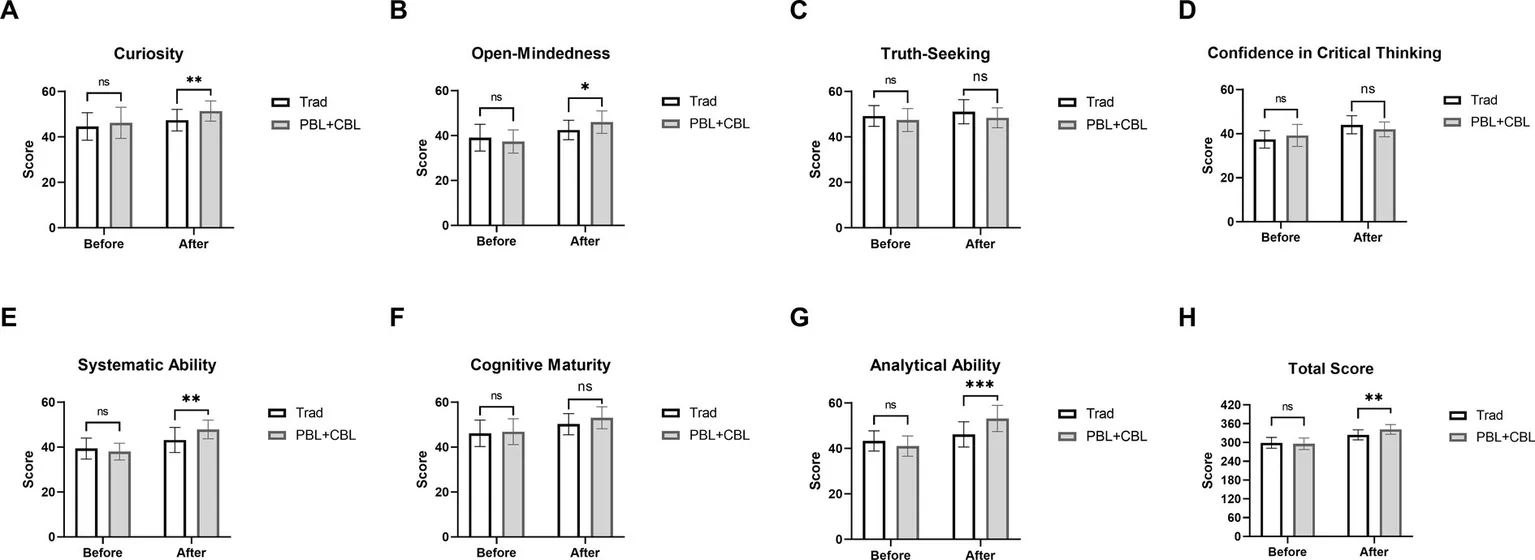
Comparison of CTDI-CV scores between two groups of medical interns before and after teaching intervention. Observation indicators included seven dimensions in **(A)** curiosity, **(B)** open-mindedness, **(C)** truth-seeking, **(D)** confidence in critical thinking, **(E)** systematic ability, **(F)** cognitive maturity, **(G)** analytical ability, and **(H)** total score. Statistical analysis was performed using Student’s *t*-test. All data are presented as mean ± standard deviation. *, *p* < 0.05; **, *p* < 0.01; ***, *p* < 0.001; ns, not significant; Trad, traditional teaching method in control group; PBL + CBL, combined PBL-CBL teaching method guided by Thorndike’s Learning Theory in experimental group.

As shown in Figure 3, prior to the teaching intervention, no statistically significant differences were observed between the two groups of students in their scores on any dimension of the CTDI-CV scale, including curiosity (*t* = −0.783, *p* = 0.439), open-mindedness (*t* = 0.963, *p* = 0.341), truth-seeking (*t* = 1.184, *p* = 0.244), confidence in critical thinking (*t* = −1.265, *p* = 0.214), systematicity (*t* = 1.010, *p* = 0.319), cognitive maturity (*t* = −0.407, *p* = 0.686), analytical ability (*t* = 1.595, *p* = 0.119), and the total score (*t* = 0.520, *p* = 0.606). However, after the teaching intervention, students in the experimental group got higher scores in the dimensions of curiosity (*t* = −2.758, *p* = 0.009), open-mindedness (*t* = −2.377, *p* = 0.023), systematicity (*t* = −3.025, *p* = 0.004), analytical ability (*t* = −3.911, *p* < 0.001), and total score (*t* = −3.476, *p* = 0.001). All these differences were statistically significant.

### 3.3 The combined PBL-CBL teaching method guided by Thorndike’s Learning Theory minimizes the medical interns’ emotional exhaustion but promotes their sense of personal achievement

**Figure 4:**
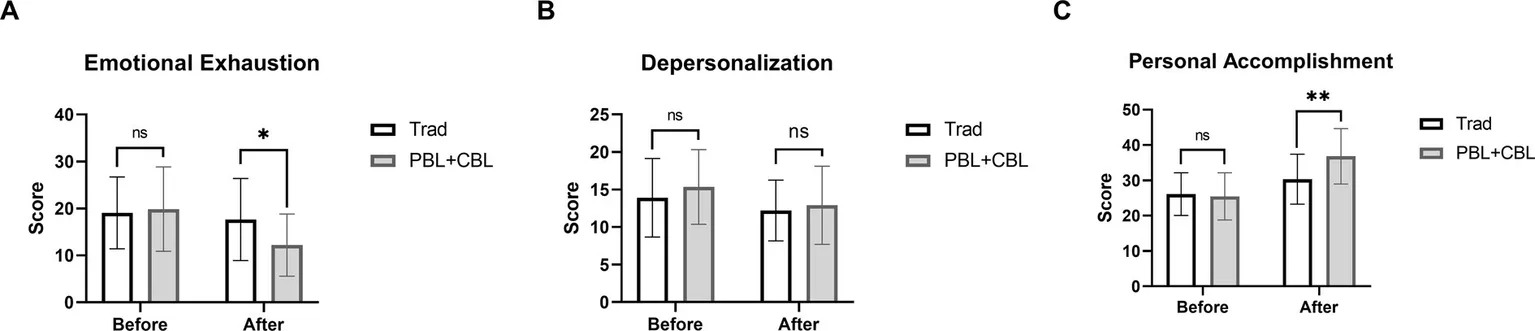
Comparison of MBI scores between two groups of medical interns before and after teaching intervention. Observation indicators included scores in **(A)** emotional exhaustion, **(B)** depersonalization, and **(C)** personal accomplishment. Statistical analysis was performed using Student’s *t*-test. All data are presented as mean ± standard deviation. *, *p* < 0.05; **, *p* < 0.01; ns, not significant; rad, traditional teaching method in control group; PBL + CBL, combined PBL-CBL teaching method guided by Thorndike’s Learning Theory in experimental group.

As shown in Figure 4, prior to the teaching intervention, no statistically significant differences were observed between the two groups of students in their scores on any dimension of the MBI scale, including emotional exhaustion (*t* = −0.304, *p* = 0.763), depersonalization (*t* = −0.899, *p* = 0.374), and personal accomplishment (*t* = 0.323, *p* = 0.748). However, after the teaching intervention, compared with the control group, students in the experimental group got lower scores of emotional exhaustion (*t* = 2.223, *p* = 0.032) while higher scores of personal accomplishment (*t* = −2.756, *p* = 0.009). Both differences were statistically significant.

### 3.4 The combined PBL-CBL teaching method guided by Thorndike’s Learning Theory receives position feedback from medical interns

**Figure 5:**
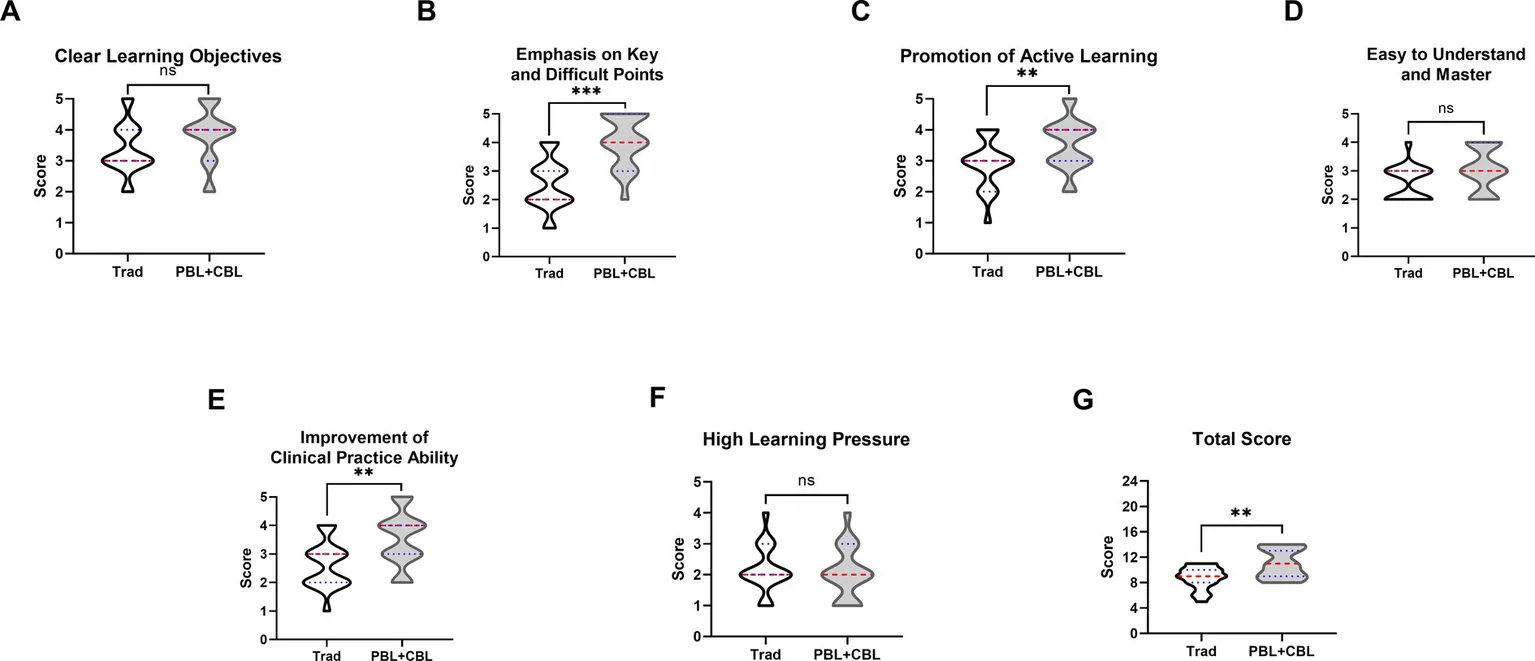
Comparison of teaching effects between two groups of medical interns before and after teaching intervention. Observation indicators included scores in **(A)** clear learning objectives, **(B)** emphasis on key and difficult points, **(C)** promotion of active learning, **(D)** easy to understand and master, **(E)** improvement of clinical practice ability, **(F)** high learning pressure, and **(G)** total score. Statistical analysis was performed using the Mann–Whitney U test. All data are presented as median (lower quartile, upper quartile). The red and blue dashed lines represent the median and interquartile range, respectively. **, *p* < 0.01; ***, *p* < 0.001; ns, not significant; Trad, traditional teaching method in control group; PBL + CBL, combined PBL-CBL teaching method guided by Thorndike’s Learning Theory in experimental group.

As shown in Figure 5, after the teaching intervention, no statistically significant differences were observed between the two groups of students in their recognition of “clear learning objectives” (*Z* = −1.806, *p* = 0.071), “easy to understand and master” (*Z* = −1.927, *p* = 0.054), and “high learning pressure” (*Z* = −0.222, *p* = 0.824). However, students in the experimental group demonstrated significantly higher ratings than the control group in “emphasis on key and difficult points” (*Z* = −4.125, *p* < 0.001), “promotion of active learning” (*Z* = −2.361, *p* = 0.018), “improvement of clinical practice ability” (*Z* = −2.707, *p* = 0.007), and “total scores” (*Z* = −2.758, *p* = 0.006). All these differences were statistically significant.

## 4 Discussion

With the advent of the post-pandemic era, public awareness of health management has reached an unprecedented level, leading to a growing demand for the detection of unknown pathogens (14). In response to various emerging infectious diseases, Medical Laboratory Technology, which serves as the “sentry” in clinical healthcare, is facing new missions and challenges in its talent cultivation. Clinical microbiology is an indispensable and core discipline in the major of Medical Laboratory Technology. It is not only the “gold standard” for diagnosing infectious diseases but also serves as a navigator for guiding the rational use of antimicrobial agents (15, 16). The importance of its teaching quality has now risen to a strategic level, crucial for safeguarding national public health security and achieving precision medicine (17, 18).

This study is the first case to apply the combined PBL-CBL teaching method guided by Thorndike’s Learning Theory (hereinafter referred to as “the combination teaching method”) to the teaching of bacterial resistance detection in the discipline of clinical microbiology. Compared with the traditional lecture-based teaching method, we found that the combination teaching method significantly improved the medical interns’ professional knowledge, cultivated their critical thinking skills, reduced their learning burnout, and finally demonstrated positive teaching effectiveness. Through in-depth analysis of the results, we identified the following key insights:

Firstly, the success of the combination teaching method lies in its full implementation of Thorndike’s Law of Readiness. Traditional teaching often leaves students in a passive receptive state, resulting in insufficient learning preparation. On the contrary, in the class using the combination teaching method, the PBL session uses real presentations of antimicrobial susceptibility reports or infection cases as “stimuli,” which effectively stimulates students’ curiosity and inquiry mindset, putting their nervous systems in a “ready” state. The activation of this internal motivation lays a crucial psychological foundation for the effective input and construction of knowledge, which explains why students in the experimental group exhibited a significant improvement in the “curiosity” dimension in the CTDI-CV scale.

Secondly, Thorndike’s Law of Exercise is reflected in the combination teaching method through multi-level and closed-loop practice. Bacterial resistance detection is a skill that highly emphasizes practice and logical reasoning. The PBL + CBL combination model provides students with a complete “stimulus–response” connection chain, encompassing “problem identification → hypothesis generation → literature research → case analysis → simulated operation → conclusion drawing.” Students engage not only in repeated mental “exercise” at the theoretical level through PBL discussions but also in skill exercise within simulated or real CBL operational environments. This repeated reinforcement through “learning by doing” solidifies key “connections,” such as standardized procedures for AST, interpretation of the AST results, and reasoning of drug resistance mechanisms. It deepens knowledge from short-term memory to long-term memory and muscle memory, which is reflected in the higher professional knowledge scores achieved by medical interns from the experimental group.

Thirdly, Thorndike’s Law of Effect is key to maintaining learning motivation and shaping correct clinical thinking. In traditional teaching, student learning effects are often only reflected through final exam scores, which are delayed and unclear. In this study, however, the combination teaching method established an immediate and diverse feedback system. In PBL group discussions, peer recognition and teacher guidance serve as positive “rewards.” In CBL operations, the sense of accomplishment from successfully identifying drug-resistant bacteria and reasonably explaining the relationship between antimicrobial susceptibility phenotypes and genotypes was the most direct “satisfaction.” Meanwhile, the formative assessments of teaching quality were implemented throughout the entire process. These positive “effects” continuously reinforce students’ correct behaviors and thinking paths, while weakening imprudent or incorrect operations and inferences, thereby effectively shaping their scientific and standardized clinical diagnostic thinking habits.

The “combination” integration of PBL and CBL is not a simple combination but forms a complementary synergy under the framework of Thorndike’s theory. PBL focuses on training students to identify, define, and explore unknown problems, while CBL emphasizes applying knowledge to solve clinical problems in given scenarios. The former develops divergent thinking and information literacy, while the latter strengthens convergent thinking and decision-making skills (19). They alternate in the cycle of “readiness-exercise-effect,” jointly forming a learning journey characterized by a multi-dimensional spiral improvement of “knowledge-skills-competency” (20, 21). This methodology not only serves as the core mechanism for students to integrate theoretical knowledge and train clinical thinking but also optimizes learning efficiency to the greatest extent and effectively reduces learning burnout.

Admittedly, this study has limitations. For instance, the samples were sourced from a single medical education institution, with a limited sample size. The research scope will be expanded to include multi-center data to enhance the generalizability of results in the future. Furthermore, the long-term follow-up evaluation of the participants was insufficient in the current study. The impact of the combination teaching method on future career competencies, such as the accuracy of clinical decision-making and the research potential, requires further investigation.

In addition, we note that while Thorndike’s laws provided useful operational guidance for our teaching design, future studies may benefit from integrating PBL and CBL with more epistemologically aligned theories such as constructivism or experiential learning theory. We also suggest that a combined theoretical framework—drawing on both behaviorist principles for structuring practice and feedback, and constructivist principles for fostering deep understanding—may offer a more comprehensive approach to medical education.

In summary, the combined PBL-CBL teaching method guided by Thorndike’s Learning Theory effectively strengthens the “stimulus–response” connections of students in learning bacterial resistance detection through well-designed learning scenarios, repeated and diverse exercises, and immediate positive feedback, thereby significantly improving teaching quality. This model not only teaches knowledge and skills but also meets the core requirements for cultivating Medical Laboratory Technology professionals in the new era. In the future, we will try to integrate artificial intelligence-assisted systems and virtual simulation technology into the combination teaching to provide more personalized “exercise” pathways and smart “effect” feedback, further deepening teaching reform.

## Data Availability

The raw data supporting the conclusions of this article will be made available by the authors, without undue reservation.
