# Supplementary material for: Evaluation of the combined PBL-CBL teaching method based on Thorndike’s Learning Theory in the education of bacterial resistance detection
Source: Front Med (Lausanne). 2026 Aug 25;13:1897289. doi: 10.3389/fmed.2026.1897289 (PMC13549919; doi:10.3389/fmed.2026.1897289)
Supplement: Supplementary file 1 [file Data_sheet_1.docx]

Supplementary Material

Evaluation of the combined PBL-CBL teaching method based on Thorndike's Learning Theory in the education of bacterial resistance detection

Yifeng Liu^1,2^, Cuiju Mo^1,2^, Meng Li^1,2*^

^1^ Department of Clinical Laboratory, The First Affiliated Hospital of Guangxi Medical University, Nanning, China

^2^ Teaching and Research Section of Clinical Laboratory Science, The First School of Clinical Medicine, Guangxi Medical University, Nanning, China

*** Correspondence:**
Meng Li
gxmulimeng@foxmail.com.

**Supplementary Table 1. Questionnaire for evaluating teaching effectiveness**

**Instruction:**

This questionnaire is designed to evaluate your perception of the teaching method you have just experienced. Please read each statement carefully and circle the number that best reflects your agreement, based on your genuine feelings. All responses are anonymous and will be used solely for research purposes. There are no right or wrong answers.

**Scoring:**

1 = Strongly disagree

2 = Disagree

3 = Neutral

4 = Agree

5 = Strongly agree

| **Item No.** | **Questionnaire item** | **Score** |
| --- | --- | --- |
| 1 | The learning objectives of this bacterial antimicrobial resistance testing practicum were clearly communicated. |  |
| 2 | The teaching method clearly emphasized the key concepts and difficult aspects of bacterial antimicrobial resistance testing. |  |
| 3 | The teaching method encouraged me to actively participate in learning, including asking questions, discussing cases, and seeking relevant information. |  |
| 4 | The teaching content was easy to understand and helped me master the relevant knowledge and practical skills. |  |
| 5 | The teaching method improved my ability to apply bacterial antimicrobial resistance testing knowledge and results in clinical practice scenarios. |  |
| 6 | This teaching method imposed a high level of learning pressure on me. |  |

**Supplementary Table 2. Tests for normality and homoscedasticity used in this study**

| **Variable** | **Before or after teaching intervention** | **Group** | **W statistic** | ***P* value of Shapiro-Wilk test** | **Normal distribution (Y/N)** | **Levene statistic** | ***P* value of Levene's test** | **Homoscedasticity (Y/N)** |
| --- | --- | --- | --- | --- | --- | --- | --- | --- |
| Theoretical knowledge | Before | Control | 0.980 | 0.938 | Y | 0.544 | 0.465 | Y |
|  |  | Experimental | 0.961 | 0.557 | Y |  |  |  |
|  | After | Control | 0.909 | 0.061 | Y | 0.205 | 0.654 | Y |
|  |  | Experimental | 0.965 | 0.652 | Y |  |  |  |
| Practical skills | Before | Control | 0.968 | 0.718 | Y | 0.100 | 0.754 | Y |
|  |  | Experimental | 0.944 | 0.280 | Y |  |  |  |
|  | After | Control | 0.978 | 0.911 | Y | 0.973 | 0.330 | Y |
|  |  | Experimental | 0.964 | 0.633 | Y |  |  |  |
| Analysis of AST reports | Before | Control | 0.962 | 0.591 | Y | 0.577 | 0.452 | Y |
|  |  | Experimental | 0.985 | 0.977 | Y |  |  |  |
|  | After | Control | 0.952 | 0.404 | Y | 0.145 | 0.706 | Y |
|  |  | Experimental | 0.938 | 0.224 | Y |  |  |  |
| Curiosity | Before | Control | 0.985 | 0.979 | Y | 0.115 | 0.736 | Y |
|  |  | Experimental | 0.951 | 0.382 | Y |  |  |  |
|  | After | Control | 0.942 | 0.256 | Y | 0.012 | 0.914 | Y |
|  |  | Experimental | 0.974 | 0.837 | Y |  |  |  |
| Open-mindedness | Before | Control | 0.957 | 0.477 | Y | 0.268 | 0.607 | Y |
|  |  | Experimental | 0.991 | 0.999 | Y |  |  |  |
|  | After | Control | 0.982 | 0.955 | Y | 1.259 | 0.269 | Y |
|  |  | Experimental | 0.966 | 0.663 | Y |  |  |  |
| Truth-seeking | Before | Control | 0.934 | 0.187 | Y | 0.171 | 0.681 | Y |
|  |  | Experimental | 0.977 | 0.895 | Y |  |  |  |
|  | After | Control | 0.981 | 0.943 | Y | 0.613 | 0.439 | Y |
|  |  | Experimental | 0.965 | 0.652 | Y |  |  |  |
| Confidence in critical thinking | Before | Control | 0.973 | 0.818 | Y | 1.629 | 0.210 | Y |
|  |  | Experimental | 0.964 | 0.618 | Y |  |  |  |
|  | After | Control | 0.979 | 0.915 | Y | 0.683 | 0.414 | Y |
|  |  | Experimental | 0.925 | 0.121 | Y |  |  |  |
| Systematic ability | Before | Control | 0.980 | 0.939 | Y | 0.876 | 0.355 | Y |
|  |  | Experimental | 0.984 | 0.976 | Y |  |  |  |
|  | After | Control | 0.982 | 0.958 | Y | 1.799 | 0.188 | Y |
|  |  | Experimental | 0.958 | 0.513 | Y |  |  |  |
| Cognitive maturity | Before | Control | 0.979 | 0.925 | Y | 0.001 | 0.974 | Y |
|  |  | Experimental | 0.980 | 0.931 | Y |  |  |  |
|  | After | Control | 0.945 | 0.304 | Y | 0.249 | 0.621 | Y |
|  |  | Experimental | 0.943 | 0.274 | Y |  |  |  |
| Analytical ability | Before | Control | 0.947 | 0.319 | Y | 0.113 | 0.739 | Y |
|  |  | Experimental | 0.968 | 0.722 | Y |  |  |  |
|  | After | Control | 0.913 | 0.074 | Y | 0.108 | 0.744 | Y |
|  |  | Experimental | 0.912 | 0.070 | Y |  |  |  |
| Total score of CTDI-CV scale | Before | Control | 0.954 | 0.431 | Y | 0.005 | 0.946 | Y |
|  |  | Experimental | 0.958 | 0.508 | Y |  |  |  |
|  | After | Control | 0.953 | 0.410 | Y | 0.036 | 0.851 | Y |
|  |  | Experimental | 0.945 | 0.300 | Y |  |  |  |
| Emotional exhaustion | Before | Control | 0.986 | 0.986 | Y | 0.819 | 0.371 | Y |
|  |  | Experimental | 0.971 | 0.775 | Y |  |  |  |
|  | After | Control | 0.924 | 0.116 | Y | 3.344 | 0.075 | Y |
|  |  | Experimental | 0.933 | 0.180 | Y |  |  |  |
| Depersonalization | Before | Control | 0.976 | 0.865 | Y | 0.027 | 0.870 | Y |
|  |  | Experimental | 0.924 | 0.116 | Y |  |  |  |
|  | After | Control | 0.938 | 0.217 | Y | 3.241 | 0.080 | Y |
|  |  | Experimental | 0.944 | 0.291 | Y |  |  |  |
| Personal accomplishment | Before | Control | 0.978 | 0.912 | Y | 0.025 | 0.876 | Y |
|  |  | Experimental | 0.988 | 0.994 | Y |  |  |  |
|  | After | Control | 0.974 | 0.828 | Y | 0.618 | 0.437 | Y |
|  |  | Experimental | 0.951 | 0.377 | Y |  |  |  |
| Clear learning objectives | NA | Control | 0.843 | 0.004 | N | NA | | NA |
|  |  | Experimental | 0.860 | 0.008 | N |  |  |  |
| Emphasis on key and difficult points | NA | Control | 0.884 | 0.021 | N | NA | | NA |
|  |  | Experimental | 0.850 | 0.005 | N |  |  |  |
| Promotion of active learning | NA | Control | 0.846 | 0.005 | N | NA | | NA |
|  |  | Experimental | 0.875 | 0.015 | N |  |  |  |
| Easy to understand and master | NA | Control | 0.744 | 0.000 | N | NA | | NA |
|  |  | Experimental | 0.816 | 0.002 | N |  |  |  |
| Enhancement of clinical practice ability | NA | Control | 0.868 | 0.011 | N | NA | | NA |
|  |  | Experimental | 0.891 | 0.028 | N |  |  |  |
| High learning pressure | NA | Control | 0.846 | 0.005 | N | NA | | NA |
|  |  | Experimental | 0.856 | 0.007 | N |  |  |  |
| Total score of teaching effects evaluation scale | NA | Control | 0.819 | 0.003 | N | NA | | NA |
|  |  | Experimental | 0.886 | 0.023 | N |  |  |  |

Abbreviations: NA, not available; Y, yes; N, no.
